# A combined analysis of multi-omics data reveals the prognostic values and immunotherapy response of LAG3 in human cancers

**DOI:** 10.1186/s40001-023-01583-9

**Published:** 2023-12-19

**Authors:** Jinwu Peng, Zhihao Du, Yuwei Sun, Zhiyang Zhou

**Affiliations:** 1 Department of Pathology, Xiangya Hospital, Central South University, Changsha, 410008 Hunan China; 2 Department of Pathology, Xiangya Changde Hospital, Changde, 415000 Hunan China; 3 National Clinical Research Center for Geriatric Disorders, Xiangya Hospital, Central South University, Changsha, 410008 Hunan China; 4 Department of Breast Surgery, Xiangya Hospital, Central South University, Changsha, 410008 Hunan China

**Keywords:** Lymphocyte-activation gene 3, Expression, Prognosis, Immune, Cancer

## Abstract

Lymphocyte-activation gene 3 (LAG3) is a highly anticipated immune checkpoint in the context of cancer, exerting regulatory control over immune cell proliferation and function to reinforce the advancement of cancers. However, the comprehensive functional analysis of LAG3 across various cancer types remains undisclosed; thus, this study aims to investigate the pan-cancer expression profile of LAG3. We have investigated the expression profile, prognostic significance, and genetic alterations of LAG3 in various cancers while elucidating its characteristic in immune response regulation. Our findings demonstrated that elevated LAG3 expression is significantly associated with favorable prognosis in patients with cutaneous melanoma (SKCM), and it may be a potential biomarker for SKCM. Furthermore, multiple immune algorithms have highlighted the important regulatory role of LAG3 for the tumor-infiltrating immune cells including CD8 + T cells, B cells, dendritic cells (DCs), macrophages, and natural killer (NK) cells. We also examined the distribution of LAG3 at the single-cell level and explored its functional significance. A comprehensive and systematic analysis of LAG3 would facilitate a comprehensive evaluation of LAG3 in cancer biology and provide valuable insights for cancer management.

**Supplementary Information:**

The online version contains supplementary material available at 10.1186/s40001-023-01583-9.

## Introduction

The abnormal changes in genes have been proved to participate in the occurrence and development of cancer. Exploring the cancer-related genes and their signaling pathways by pan-cancer analysis could contribute to the diagnosis and clinical treatment of human cancers [1, 2].

Lymphocyte-activation gene 3 (LAG3) is located on chromosome 12 (12p13.32) and encodes a type 1 transmembrane protein involved in tumor immune regulation and clinical aggressiveness [3, 4]. It is widely expressed on various immune cells, predominantly found on the surface of diverse T lymphocytes including CD4 + and CD8 + subsets, as well as other immune cell populations [5]. As an inhibitory receptor, LAG3 exerts its influence on tumor progression by modulating T cell activation and effector function [6]. However, as an immunosuppressive receptor, there is a complex interplay between LAG3 and the infiltration and functionality of immune cells. In breast cancer, elevated levels of LAG3 expression have been positively associated with multiple immune cell infiltrates [7]. The immunohistochemical staining in gastric cancer reveals a positive correlation between an increased number of LAG3-positive cells and patient prognosis [8]. Furthermore, LAG3 can also stimulate mature dendritic cells (DCs) to produce cytokines such as IL-12 and TNF-a [34067904]. Notably, the emerging animal experiments and clinical trials have demonstrated that blockade of LAG3 enhances the anti-tumor efficacy, underscoring its potential in cancer therapy [9–11]. Given the intricate expression pattern of LAG3 across different cancers, comprehensive exploration of this receptor from a pan-cancer perspective is imperative.

In this study, we comprehensively assessed the characteristics of LAG3 in various cancer types using a range of bioinformatics platforms. We conducted comparative analyses of differential expression profiles and gene alterations of LAG3 between cancer and normal tissues, and evaluated their prognostic significance on the prognostic values and immune responses.

## Materials and methods

### Gene expression analysis

We conducted an analysis of LAG3 expression profiles between tumor tissues and normal tissues using Tumor Immune Estimation Resource 2.0 (TIMER2.0). We utilized the Gene Expression Profiling Interactive Analysis 2 (GEPIA2) database to investigate the relationship between LAG3 expression and the pathological stage of TCGA tumors.

### Immumohistochemical staining

20 pairs of paraffin-embedded tissues from patients diagnosed with cutaneous malignant melanoma or non-specific skin inflammation were included. The LAG3 rabbit-derived polyclonal antibody from Proteintech Group (29,548-1-AP) was utilized with a dilution ratio of 1:4000 for this experiment.

### Genetic alteration evaluation

The cBio Cancer Genomics Portal (cBioPortal) an interactive algorithm containing multidimensional cancer genomics datasets, was employed to investigate the molecular profiles and clinical attributes of pan-cancer samples in terms of genomics. In this study, the cBioPortal platform was utilized to evaluate the genetic alterations of LAG3 in TCGA cancers, including the types of genetic alteration and mutation sites.

### Survival analysis

The prognostic value of LAG3 expression in multiple cancers was assessed using the GEPIA2 database. Additionally, the cBioPortal platform was utilized to determine the survival significance of LAG3 genetic alterations in cancer patients, encompassing overall survival (OS), disease-free survival (DFS), disease-specific survival (DSS) and progression-free survival (PFS).

### Immune infiltration analysis

The TIMER2.0 software was employed to investigate the correlation between LAG3 expression and various tumor-infiltrating immune cells populations, including B cells, CD8 + T cells, DCs, macrophages, neutrophils, natural killer cells (NK cells) and monocytes. Furthermore, the Gene Set Enrichment Analysis (GSEA) algorithm available at Xiantao XueShu (https://www.xiantao.love/products) was utilized to validate the roles of LAG3 in the regulation of immune-associated signaling pathways.

### LAG3-associated gene enrichment analysis

The STRING was utilized to construct the LAG3-associated molecule network. We applied the GEPIA2 database to investigate the top 100 LAG3-related molecules in TCGA pan-cancer dataset. Subsequently, Pearson correlation analysis was performed to assess the relationship between LAG3 and the top 6 LAG3-related molecules across various cancer types. GSEA enrichment analysis was conducted to elucidate the underlying biological functions of LAG3 in cancer progression.

### Single-cell level assessment of LAG3 expression

We analyzed the single-cell distribution patterns and biological functions of LAG3 using CancerSEA tool [16]. The Tumor Immunity Single Cell Center (TISCH) [17] is a scRNA-seq database specifically dedicated to investigating the tumor microenvironment (TME). We also employed TISCH database to investigate the expression pattern of LAG3 in SKCM at single-cell level.

## Results

### The expression profiles of LAG3 in pan-cancer

**Fig. 1:**
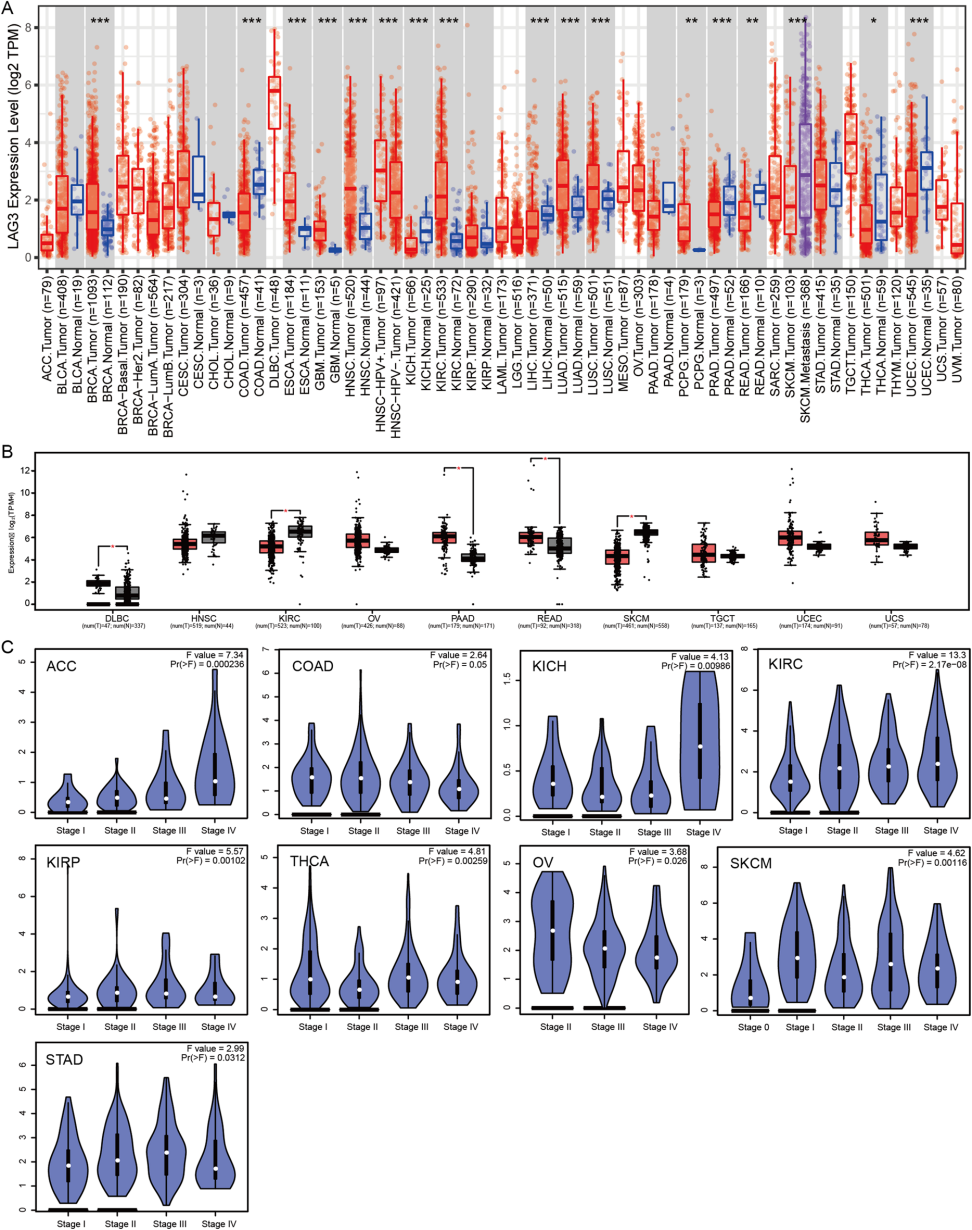
The expression levels of LAG3 in pan-cancer. **A** TIMER2.0 demonstrated the expression of LAG3 across TCGA cancers and the corresponding normal tissues. **B** The combination of TCGA and GTEx databases revealed the differential expression of LAG3 in tumor groups compared to normal groups. **C** The correlation between LAG3 expression and patients’ pathological stages were assessed in TCGA patients. ***p < 0.001; **p < 0.01 and *p < 0.05

We utilized the TIMER2.0 platform to investigate the expression profiles of LAG3 in tumor tissue and normal tissue derived from TCGA datasets, thereby revealing distinct patterns of LAG3 expression across various cancer types. The upregulated expression of LAG3 was observed in BRCA, ESCA, GBM, HNSC, KIRC, LUAD, LURC, and PCPG. The expression of LAG3 was found to be downregulated in several cancer types, including COAD, KICH, LIHC, PRAD, READ, THCA and UCEC (Fig. 1A). By integrating transcriptome data from TCGA and GTEx datasets, we further confirm the differential expression levels of LAG3 between tumor and normal tissues. Consistently, we found the upregulated expression of LAG3 in DBLC, OV, PAAD, READ, TGCT, UCEC and UCS, while downregulated expression in HNSC, KIRC and SKCM (Fig. 1B). These findings indicated the different expression profiles of LAG3 across diverse cancers types.

Subsequently, we utilized the GEPIA2 database to analyze the correlation between LAG3 expression and patients’ pathological stage. The violin plots clearly demonstrated LAG3 expression was positively associated with the stage of patients with ACC and KIRC, while negatively associated with the stage of patients with COAD and OV (Fig. 1C). These findings collectively highlighted the heterogeneous functions of LAG3 across different cancer types.

**Fig. 2:**
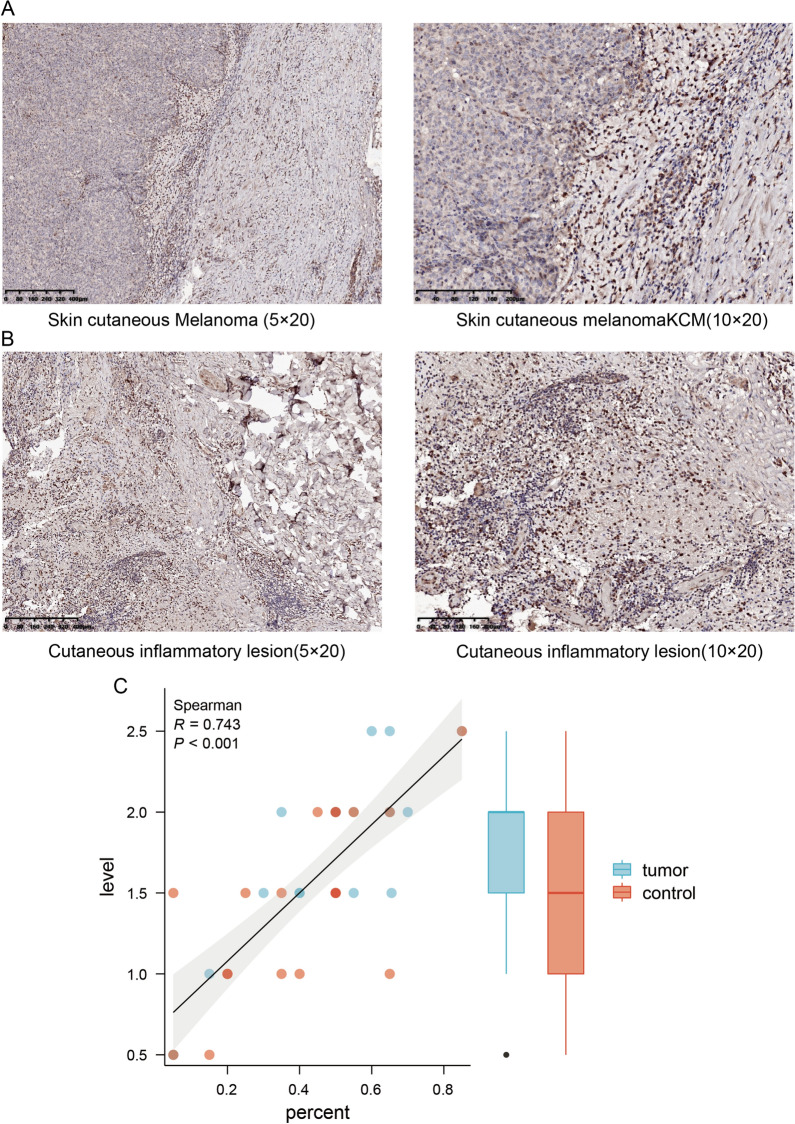
Immunohistochemical staining of LAG3 in SKCM tissues and control tissues. **A**,** B** The immunostaining of LAG3 in SKCM tissue and cutaneous inflammatory tissue was examined. **C** The relationship between LAG3 staining intensity and the ratio of LAG3-positive cells in all inflammatory cells

Next, a total of 40 paraffin-embedded skin tissues were collected, comprising 20 malignant melanoma tissues and 20 non-specific inflammatory skin tissues. As shown in Fig. 2A, B, lower levels of LAG3 expression were detected in tumor tissues compared to control tissues. In addition, LAG3 was predominantly expressed in immune cells, particularly T lymphocytes. To investigate whether the expression pattern of LAG3 was similar between malignant melanoma and inflammatory skin tissues, a hierarchical analysis of immunostaining was conducted. The staining intensity was categorized into six scores: weak expression (1.5–1.0), medium expression (1.5–2.0), and strong expression (2.5–3.0). Following meticulous evaluation, a significant positive correlation between the intensity and proportion of LAG3 staining in infiltrating immune cells from two groups examined here was observed (Fig. 2C). Our research findings supported the potential roles of LAG3 for assessing the extent of inflammation.

### Survival analysis of LAG3 in pan-cancer

**Fig. 3:**
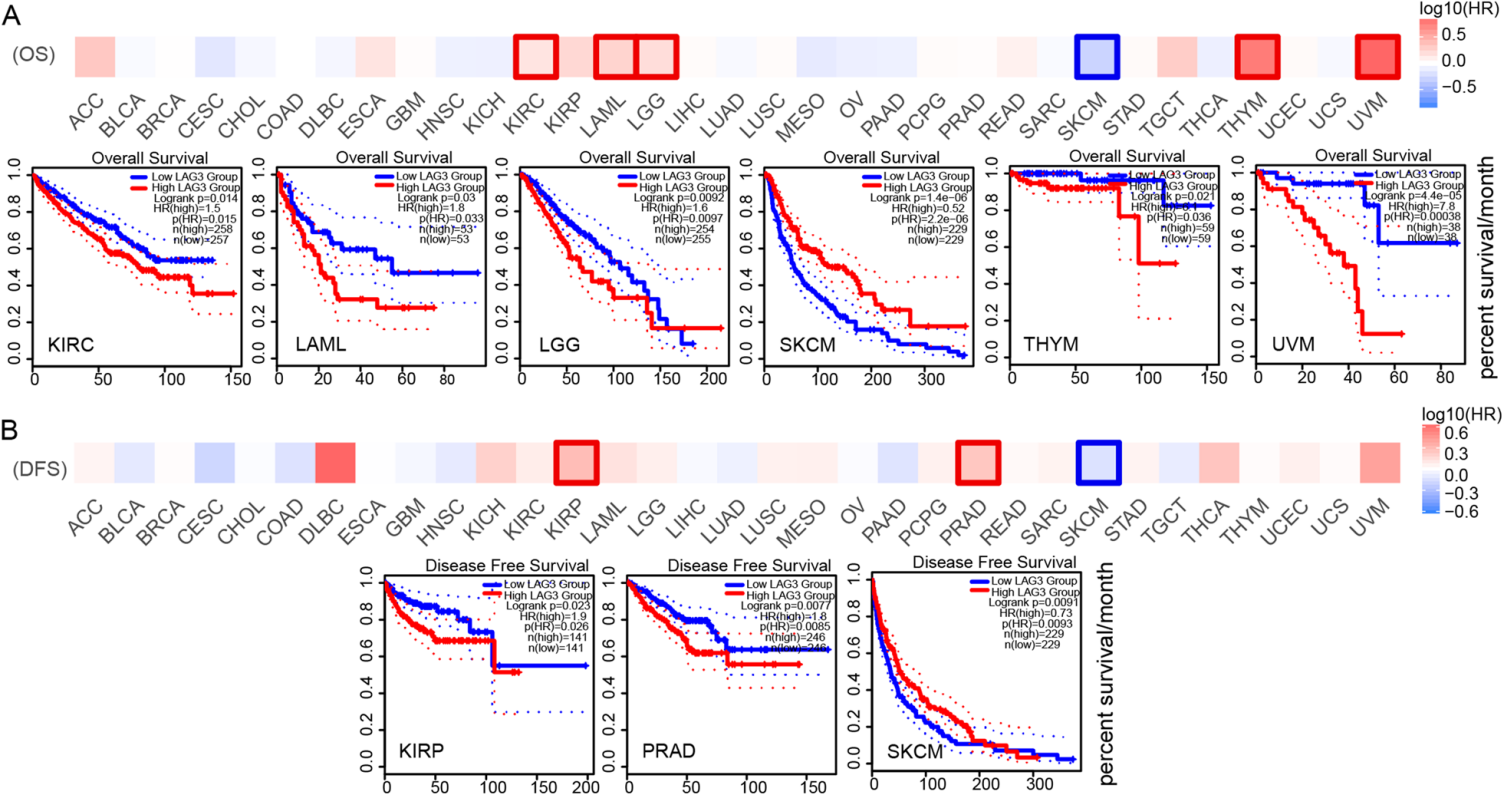
The impact of LAG3 expression on overall survival (OS) and disease-free survival (DFS) in human cancers. **A** The values of LAG3 on overall survival (OS) in pan-cancer analysis. **B** The values of LAG3 on disease-free survival (DFS) in pan-cancer analysis

We utilized the GEPIA2 database to assess the survival outcomes of LAG3 in various cancers types, focusing on OS and DFS. Our analysis revealed that higher expression of LAG3 were associated with poorer OS in KIRC, LAML, LGG, THYM and UVM patients. Conversely, elevated LAG3 expression was correlated with improved OS in SKCM patients (Fig. 3A). Additionally, higher LAG3 expression exhibited unfavorable RFS in patients with KIRP and PRAD, and favorable RFS in SKCM patients (Fig. 3B). Collectively, these findings exhibited strongly supported the tumor-suppressive roles of LAG3 in SKCM.

### The genetic alteration of LAG3 in pan-cancer

**Fig. 4:**
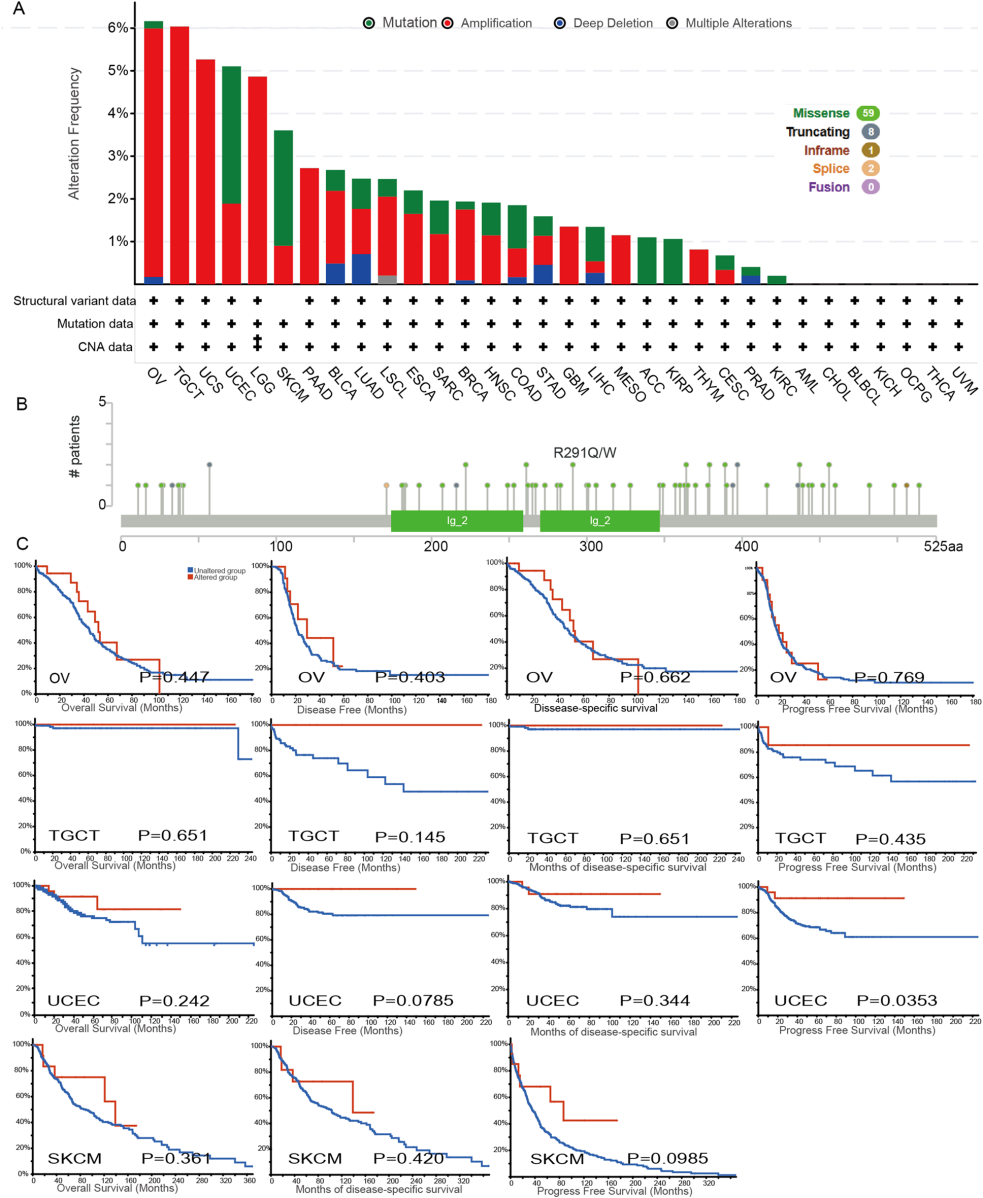
Mutation status of LAG3 in TCGA tumors was analyzed by cBioPortal. **A**,** B** The alteration frequency (**A**) and mutation site (**B**) were displayed. **C** The correlation between LAG3 mutation status and OS, DSS, DFS and PFS in patients with OV, TGCT, UCEC and SKCM

We utilized the cBioPortal tool to explore the genetic alterations of LAG3 in pan-cancer. The predominant types of LAG3 genetic alteration observed in Fig. 4A were amplification and mutation. Notably, OV, TGCT, UCS, UCEC, LGG and SKCM exhibited the highest frequency of genetic alteration. Among these cancers, TGCT, OV and UCS displayed the highest frequency of LAG3 amplification, while UCEC and SKCM had the highest frequency mutation. The identified mutation types in LAG3 primarily included missense mutation, truncating mutation, inframe mutation and splicing mutation, with missense mutations being the most prevalent type. Notably, the R291Q/W missense mutation has emerged as the predominant mutational factor, which might be a putative driver factor for the cancer pathogenesis (Fig. 4B). Furthermore, we assessed the prognostic implications of LAG3 genetic alteration in cancer patients. Our findings demonstrated that UCEC patients with LAG3 genetic alteration exhibited a poorer PFS compared to those without alteration (Fig. 4C). However, no significant associations were observed between LAG3 genetic alteration and patients’ outcomes in OV, TGCT, UCEC and SKCM. These findings suggested that LAG3 genetic alteration might affect the prognosis of cancer patients, especially UCEC patients.

### Enrichment analysis revealed the functional regulation of LAG3 in immune response

**Fig. 5:**
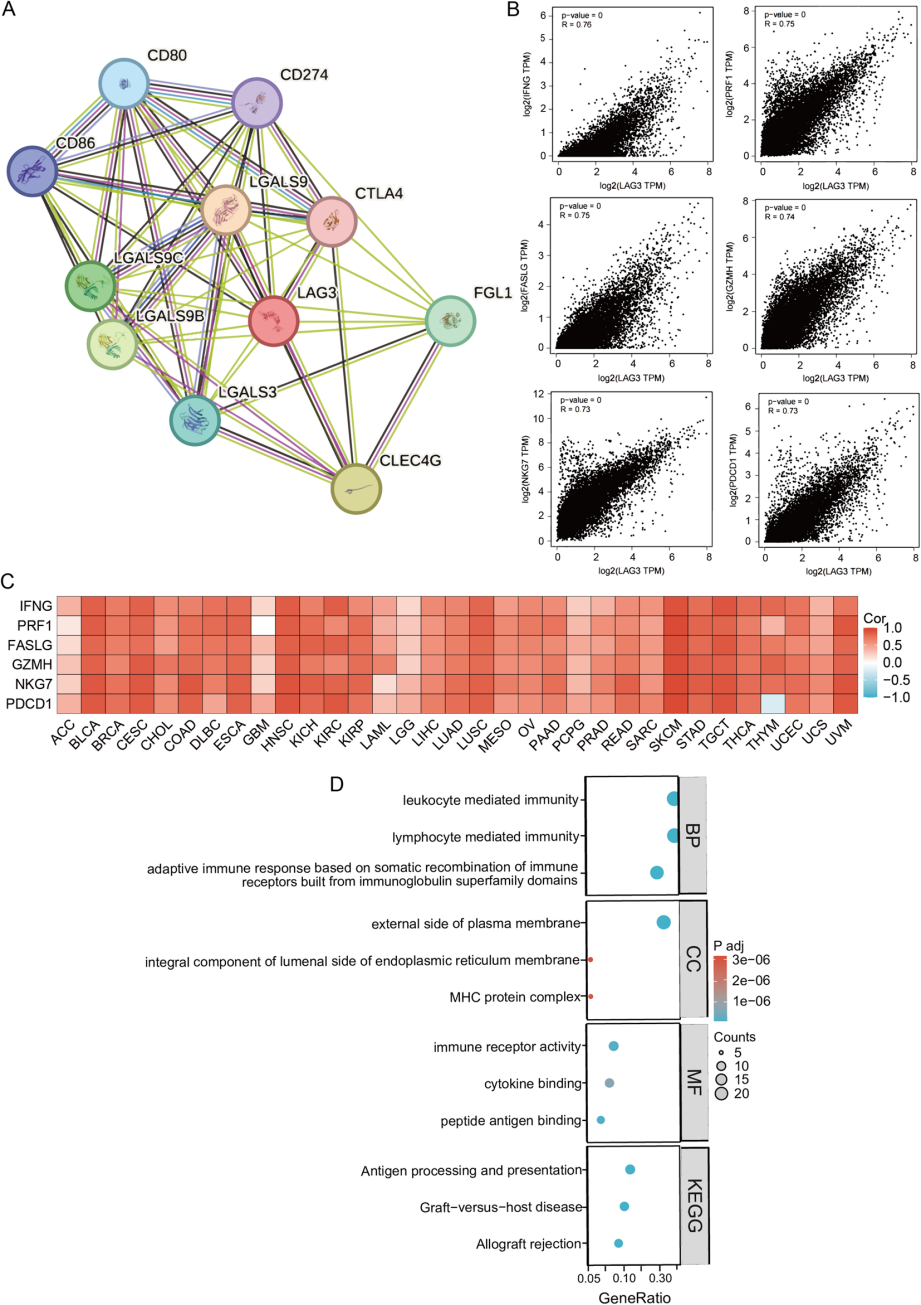
LAG3-related gene enrichment and pathway analysis. **A** STRING protein network of experimentally determined LAG3-correlated molecules. **B** Expression correlation between LAG3 and representative genes (IFNG, PRF1, FASLG, GZMH, NKG7 and PDCD1) in TCGA datasets as determined by GEPIA2. **C** Heatmap of the expression correlation between LAG3 and representative genes (IFNG, PRF1, FASLG, GZMH, NKG7 and PDCD1) in the pan-cancers. **D** GO/KEGG enrichment analysis of representative immune processes

We analyzed the functional enrichment of LAG3-related genes in various cancer types. The STRING tool was utilized to construct the molecule network of associated with LAG3 (Fig. 5A). Subsequently, we obtained the top 100 LAG3-related genes from the GEPIA2 database (Additional file 2: Table S1). Our findings revealed a positive correlation between the expression levels of LAG3 and IFNG (*R* = 0.76, *p* < 0.001), PRF1 (*R* = 0.75, *p* < 0.001), FASLG (*R* = 0.75, *p* < 0.001), GZMH (*R* = 0.74, *p* < 0.001), NKG7 (*R* = 0.74, *p* < 0.001), and PDCD1 (*R* = 0.73, *P* < 0 0.001) in pan-cancer tissues (Fig. 5B). Furthermore, the heatmap showed a consistent positive association between LAG3 and the aforementioned genes in most cancer types (Fig. 5C). Furthermore, Xiantao-based GO/KEGG enrichment analysis revealed a significant enrichment of LAG3-related genes in several immune-associated processes, such as leukocyte mediated immunity and lymphocyte mediated immunity (Fig. 5D). The enrichment analysis suggested that LAG3 and its related genes might exert the functional roles on the regulation of immune response.

### The biological function of LAG3 at the single-cell level

**Fig. 6:**
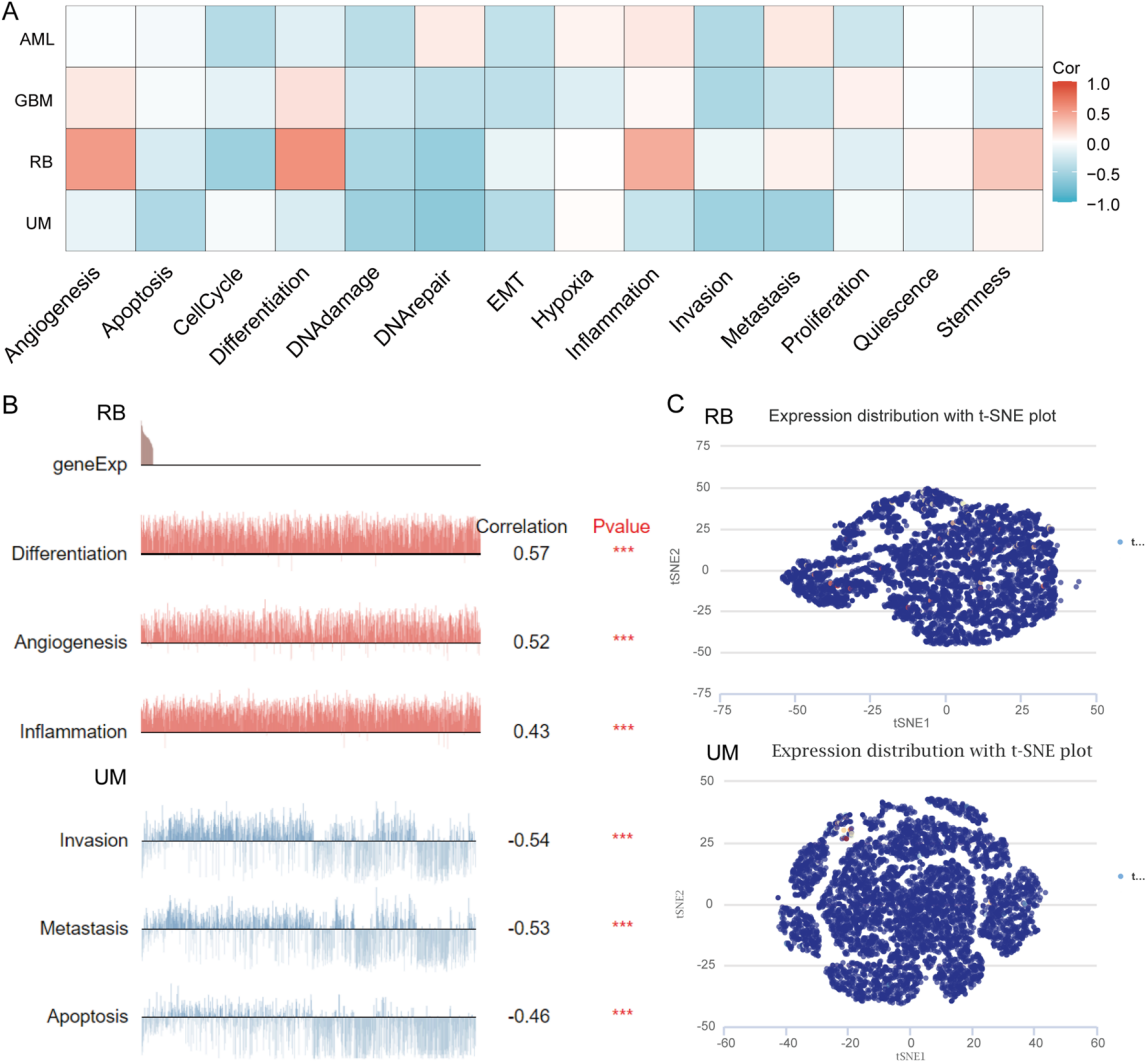
The expression levels of LAG3 at a single-cell sequence level. **A**,** B** The CancerSEA tool displayed the relationship between LAG3 expression and different functional status across pan-cancers. **C** The t-SNE diagrams portrayed the distributions of LAG3 expression at single-cell levels from RB and UM samples

**Fig. 7:**
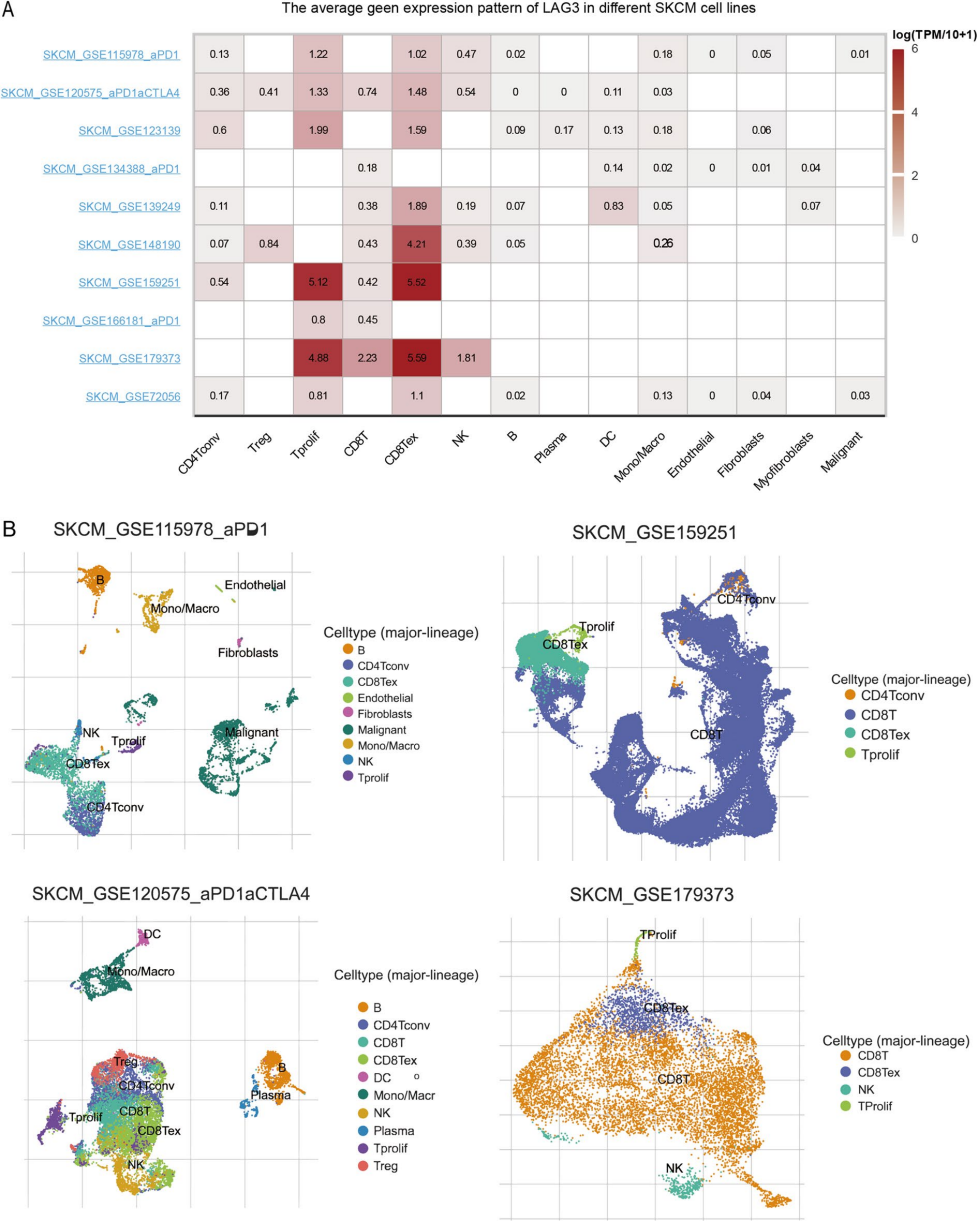
LAG3 expression at the single-cell level within tumor microenvironment of SKCM. **A** The expression of LAG3 is predominantly observed in several immune cells. **B** The distribution pattern of LAG3 at single-cell level in several SKCM datasets

The CancerSEA database was subsequently employed to investigate the significance of LAG3 at the single-cell level. Figure 6A illustrates the function status of LAG3 across various cancer types. In retinoblastoma (RB), the expression of LAG3 exhibited a positive correlation with angiogenesis and inflammation (Fig. 6B), suggesting its regulatory roles in shaping of tumor microenvironment. In uveal melanoma (UM), there was a negative correlation between LAG3 expression and invasion and metastasis (Fig. 6B). Figure 6C presents the distribution pattern of LAG3 at the single-cell level with in RB and UM samples. Next, we further investigated the LAG3 expression in various SKCM-derived cell lines using the TISCH database. The data revealed that LAG3 was present in diverse immune cell populations, particularly CD8 + T cells (Fig. 7A). The detailed distribution of LAG3 in various immune cells within several datasets is shown in Fig. 7B. These findings indicated that LAG3 might participate in modulating the CD8 + T cell response during tumorigenesis.

### The association between LAG3 expression and tumor-infiltrating immune cells

**Fig. 8:**
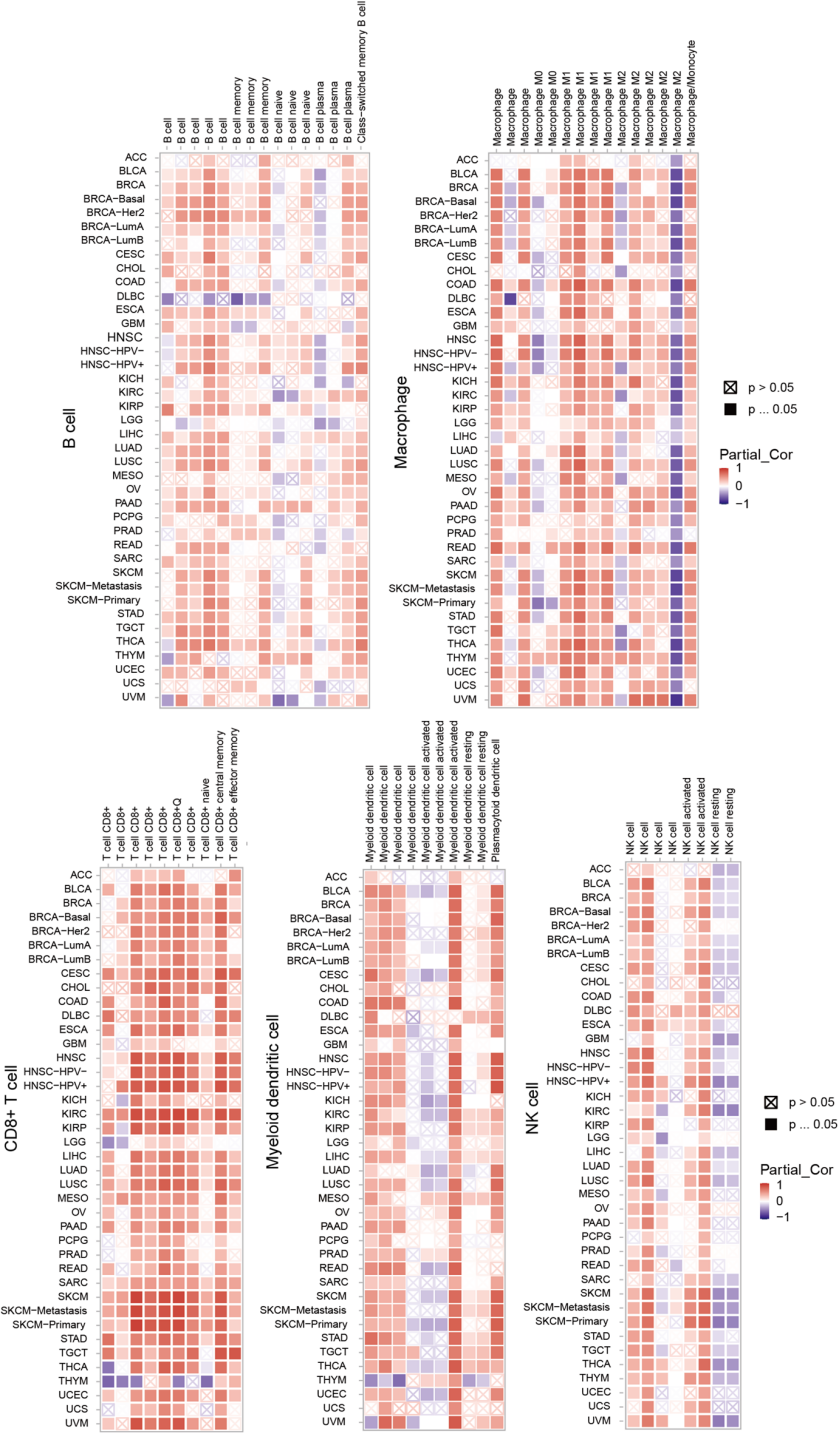
The correlation between the LAG3 expression and several immune cell lines. The TIMER2.0 database depicted the relationship between LAG3 expression and immune infiltration of CD8 + T cells, B cells, DCs, macrophage and NK cells

Based on several algorithms from TIMER2.0 database, we investigated the correlation between tumor-infiltrating immune cells and LAG3 expression across various cancer types. As shown in Fig. 8, we found a positive association between LAG3 expression and tumor infiltration of CD8 + central memory T cell in CESC, KIRC, SKCM, OV, UCEC, etc. Additionally, LAG3 expression exhibited a positive correlation with infiltration of B cell in LUAD, PAAD, OV, SKCM, TGCT and UCEC. Furthermore, LAG3 expression was positively correlated with the infiltration of activated myeloid DCs and plasmacytoid DCs in most cancers, such as BLCA, HNSC, KIRC, OV, and UCEC. Moreover, M1 macrophage infiltration demonstrated a positive correlation with LAG3 expression in several cancers including BLCA, HNSC, KIRC, READ, OV, SKCM and UCEC. Interestingly, ESCA showed a positive correlation between infiltration of NK cell and LAG3 expression while BRCA, GBM and SKCM displayed the negative correlation. Notably, no significant correlations were observed between LAG3 expression and other immune cell infiltrations (Additional file 1: Fig S1).

## Discussion

LAG3, locating on chromosome 12 (12p13.32) and encoding a type 1 transmembrane protein, has been proved to be involved in immune regulation and clinical aggressiveness of cancer [18]. In breast cancer and lung adenocarcinoma, the high expression of LAG3 is closely related to the increase of T cell infiltration [7]. LAG3 could also directly and competitively bind with pHLA-II, to inhibiting T cell activation by affecting the combination of CD4 and pHLA-II [19]. Here, we performed a comprehensive analysis of LAG3 through multiple bioinformatics platforms to explore the functional roles of LAG3 in pan-cancer.

Based on the TCGA database, we investigated the expression of LAG3 in 33 cancers. We noticed that LAG3 was upregulated in most cancers, including BRCA, ESCA, GBM, HNSC, KIRC, LUAD, LURC, PCPG and STAD. Conversely, LAG3 was downregulated in COAD, KICH, LIHC, PRAD, READ, THCA and UCEC. Meanwhile, GEPIA2 database proved that in ACC, KIRC, COAD and OV, LAG3 expression was consistent with the pathological stage of patients. Then, Kaplan–Meier plotter showed that OV and UCEC patients with high LAG3 expression had better OS and RFS, while KIRC patients with high LAG3 expression had worse prognosis. These results suggest that the aberrantly expressed LAG3 could be a potential predictor for the prognosis of UCEC and OV cancer patients, and can assist in clinic pathological staging.

In addition, we also proved that the expression of LAG3 was significantly related with immune cell infiltration, including macrophage, CD8 + T cells, B cells, DCs, macrophage and NK cells. In earlier studies, LAG3 was a negative regulator of T cell activation and function [20]. Blocking the effect of LAG3 on human CD4 clones leads to increased production of IL-2, IL-4, IFN-γ, and TNF-α [21]. In mouse models of ovarian cancer, co-blocking LAG3 and PD-1 expressed on CD4 + and CD8 + TILs induces enhanced anti-tumor response [6]. Recent studies have shown that the combination of T cell checkpoint 41BB agonist and LAG3 antagonist could improve the anti-tumor immunity in pancreatic cancer [11]. Increased expression of LAG3 driven by IL6 led to decreased function of peripheral CD8 + T cells and reduced tumor therapeutic resistance [22]. Other studies also confirmed the regulatory roles of highly expressed LAG3 in the tumor infiltration of immune cells, including T cells, B cells, NK cells and DCs [23]. These results suggested the significant regulatory roles of LAG3 in the regulation of immune response.

Recent studies showed that high expression of LAG3 predicts a poor prognosis in patients due to its promotion of immune escape [24]. However, in SKCM, high expression of LAG3 suggests a favorable prognosis. These positive effects may be attributed to LAG3-mediated hyperactivation of immune system in SKCM [25, 26]. The discovery highlights the potential of LAG3 as a biomarker for assessing the immune system status in patients with SKCM, thereby offering valuable insights for its therapeutic management.

## Conclusions

In conclusion, this is the first extensive research about the expression profiles, prognostic values, and functional significance of LAG3 in human pan-cancers. We demonstrate that LAG3 may be a novel prognostic marker for OV and UCEC. In addition, LAG3 expression could be associated with the tumor-infiltrating immune cells and might be an underlying predictive biomarker for cancer immunotherapeutic response. These results proved that LAG3 was closely related to tumor pathogenesis and immune response.

### Supplementary Information

**Additional file 1: Fig. S1.** No significant correlation could be found between LAG3 expression and tumor infiltration of neutrophils and CD4 + T cells.

**Additional file 2: Table S1.** The top 100 LAG3-related genes from the GEPIA2 database.

## Data Availability

The data used to support this article are included within the article.
