# Supplementary figures and images for: A combined analysis of multi-omics data reveals the prognostic values and immunotherapy response of LAG3 in human cancers
Source: Eur J Med Res. 2023 Dec 19;28:604. doi: 10.1186/s40001-023-01583-9 (PMC10729452; doi:10.1186/s40001-023-01583-9)

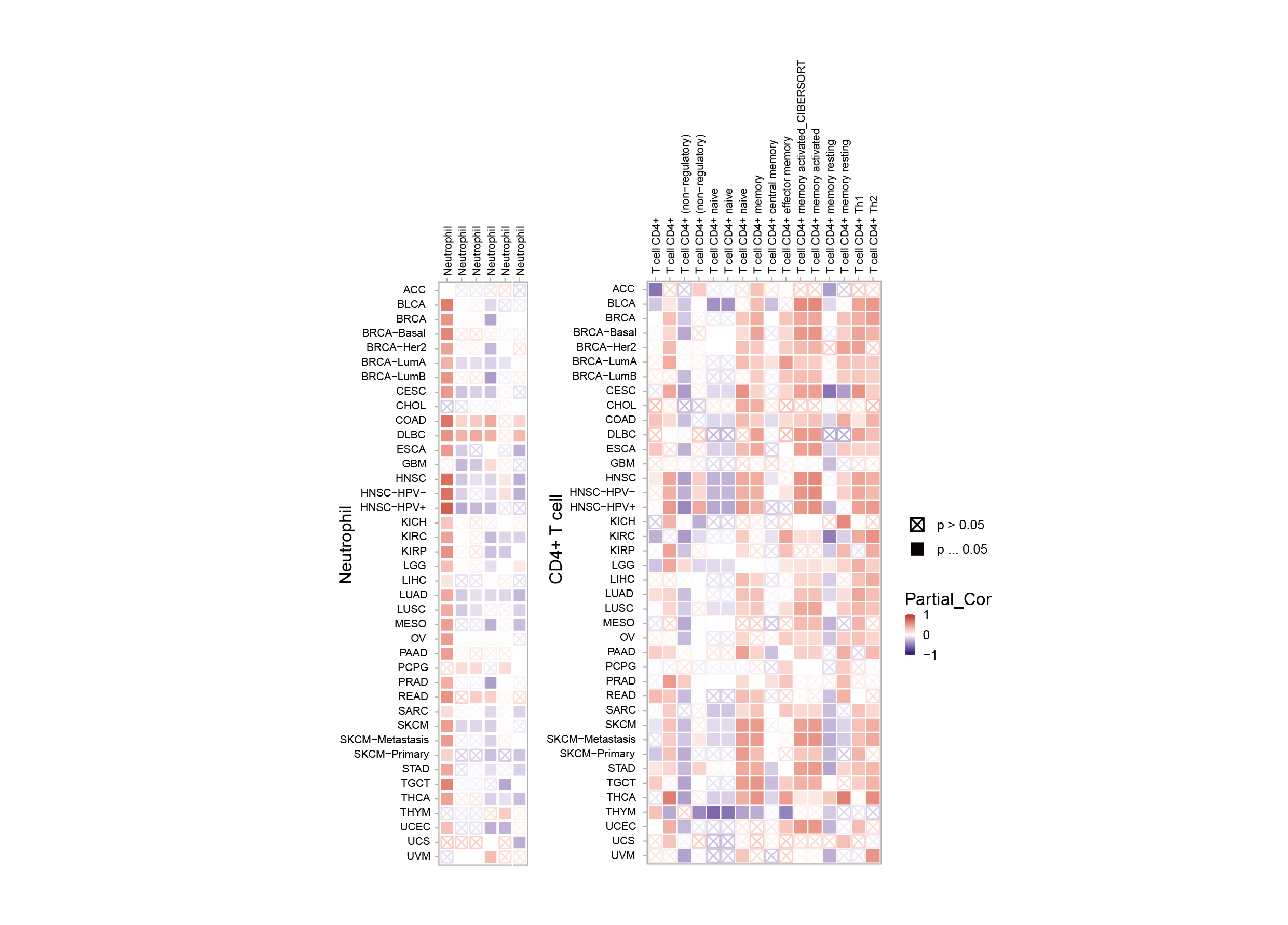

Supplement: Supplementary file 1 — Additional file 1: Fig. S1. No significant correlation could be found between LAG3 expression and tumor infiltration of neutrophils and CD4 + T cells. [file 40001_2023_1583_MOESM1_ESM.tif]
